# Cyclotron Production of Gallium-68 Radiopharmaceuticals Using the ^68^Zn(p,n)^68^Ga Reaction and Their Regulatory Aspects

**DOI:** 10.3390/pharmaceutics15010070

**Published:** 2022-12-26

**Authors:** Zarif Ashhar, Muhammad Fakhrurazi Ahmad Fadzil, Muhamad Faiz Othman, Nor Azah Yusof, Muhammad Adib Abdul Onny, Noratikah Mat Ail, Siti Fatimah Abd Rahman

**Affiliations:** 1 Pharmacy Department, National Cancer Institute, Putrajaya 62250, Malaysia; 2 Faculty of Pharmacy, Universiti Teknologi Mara, Puncak Alam 42300, Malaysia; 3 Department of Chemistry, Faculty of Science, Universiti Putra Malaysia, Serdang 43400, Malaysia; 4 Nuclear Medicine Department, National Cancer Institute, Putrajaya 62250, Malaysia; 5 School of Electrical and Electronic Engineering, Engineering Campus, Universiti Sains Malaysia, Nibong Tebal 14300, Malaysia

**Keywords:** cyclotron targetry, radiopharmaceutical, solid target, liquid target, Good Manufacturing Practices (GMP)

## Abstract

Designing and implementing various radionuclide production methods guarantees a sustainable supply, which is important for medical use. The use of medical cyclotrons for radiometal production can increase the availability of gallium-68 (^68^Ga) radiopharmaceuticals. Although generators have greatly influenced the demand for ^68^Ga radiopharmaceuticals, the use of medical cyclotrons is currently being explored. The resulting ^68^Ga production is several times higher than obtained from a generator. Moreover, the use of solid targets yields end of purification and end of synthesis (EOS) of up to 194 GBq and 72 GBq, respectively. Furthermore, experiments employing liquid targets have provided promising results, with an EOS of 3 GBq for [^68^Ga]Ga-PSMA-11. However, some processes can be further optimized, specifically purification, to achieve high ^68^Ga recovery and apparent molar activity. In the future, ^68^Ga will probably remain one of the most in-demand radionuclides; however, careful consideration is needed regarding how to reduce the production costs. Thus, this review aimed to discuss the production of ^68^Ga radiopharmaceuticals using Advanced Cyclotron Systems, Inc. (ACSI, Richmond, BC, Canada) Richmond, Canada and GE Healthcare, Wisconsin, USA cyclotrons, its related factors, and regulatory concerns.

## 1. Introduction

**Figure 1:**
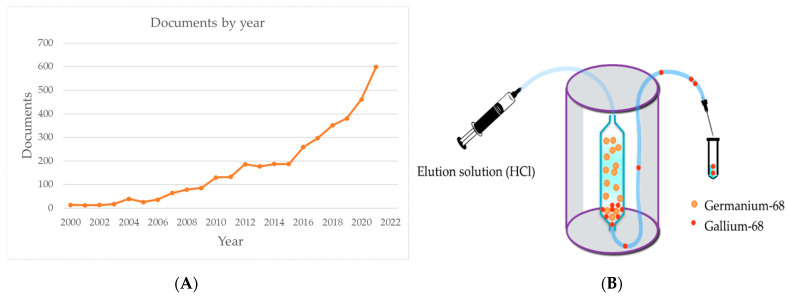
(**A**) Number of documents by year related to ^68^Ga-based on Scopus search; (**B**) Illustration of ^68^Ga radionuclide elution from ^68^Ge/^68^Ga generator using hydrochloric acid (HCl). Arrow indicating flow of eluate.

Gallium-68 (half-life 67.6 min, 89% β^+^, 830 keV) has been increasingly well-known due to its role as a radioisotope for positron emission tomography (PET) in the 2000s. The advent of the Gallium-68 (^68^Ga) generator widened the use of ^68^Ga despite its short half-life for various examinations. More importantly, its radiochemistry and chelator development has been understood. A Scopus search shows that the number of publications on ^68^Ga has exponentially increased from 2000 to 2021 (Figure 1A). Interest in ^68^Ga radiopharmaceuticals further came into the limelight with the introduction of radiopharmaceuticals for neuroendocrine tumor imaging (NET), i.e., [^68^Ga]Ga-DOTA-TOC, [^68^Ga]Ga-DOTA-NOC, and [^68^Ga]Ga-DOTA-TATE [1,2,3,4,5]. ^68^Ga-DOTA-TATE was approved by the United States Food and Drug Administration (USFDA) in 2016 [6], followed by ^68^Ga-DOTA-TOC in 2019 [7]. 

Developments in PET imaging for prostate cancer (PCa) with radiolabeled [^68^Ga]Ga-PSMA-11 have also attracted the interest of nuclear medicine physicians [8,9,10]. The product was approved in 2020 [11], making it the third ^68^Ga-based product approved by the USFDA in 5 years. The ^68^Ga-based radiopharmaceuticals have been increasingly well-known due to the superior quality of PET imaging over single photon emission computed tomography [12,13], expanding the knowledge about gallium radiochemistry and, most importantly, ^68^Germanium/^68^Gallium (^68^Ge/^68^Ga) generator availability. Recent developments combining ^68^Ga with therapeutic radionuclides such as lutetium-177 (^177^Lu) for theranostic application in both NET and PCa [14,15,16] further increase the demand for ^68^Ga-based radiopharmaceuticals. 

The main source of ^68^Ga in many nuclear medicine facilities is the ^68^Ge/^68^Ga generator (Figure 1B). The short-lived daughter isotope is eluted with hydrochloric acid, resulting in a high activity with a low ^68^Ge breakthrough. Depending on the type and age of the generator, between 0.74 GBq and 3.7 GBq of ^68^Ga activity is produced per elution, and the generator can be operated for 1 year, albeit with a limited yield [17]. In addition to the generator, the ^68^Ga produced in the cyclotron has recently received more attention due to (1) the cost and uncertainty of a constant supply of ^68^Ge/^68^Ga generators and (2) the upsurge of medical cyclotrons worldwide [18,19]. Two promising production methods using medical cyclotrons are ^68^Zn(p,n)^68^Ga and ^65^Cu(α,n)^68^Ga reactions. The first method can be performed with a small medical cyclotron [20] and provide a highly specific activity suitable for routine production and radiopeptide labeling [21,22,23].

**Figure 2:**
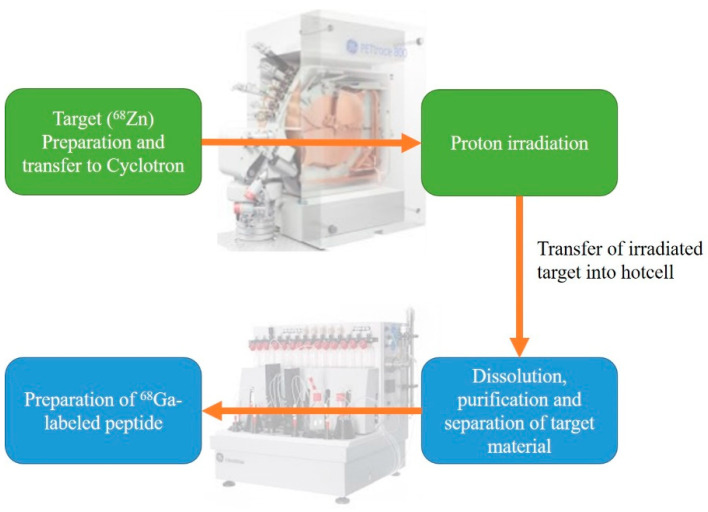
Steps in the production of the ^68^Ga-labeled peptide from a medical cyclotron.

The production of ^68^Ga-labeled peptides from a medical cyclotron (Figure 2) involves four main steps: (1) target preparation; (2) proton irradiation; (3) dissolution, purification, and separation of target material; and (4) radiolabeling of ^68^Ga-labeled peptides.

The production of ^68^Ga via the ^68^Zn(p,n)^68^Ga nuclear reaction results in the co-production of ^66^Ga and ^67^Ga [24]. Removing ^66^Ga and ^67^Ga impurities is difficult through separation techniques due to similar chemical properties and would be impractical as ^68^Ga has a short half-life. However, these impurities can be limited during irradiation with the proton irradiation energy range Ep = 14 → 5 MeV, consequently preventing ^68^Zn(p,2n)^67^Ga and ^68^Zn(p,3n)^66^Ga nuclear reactions [24]. Co-production of ^66^Ga and ^67^Ga can also exist through ^66^Zn(p,n)^66^Ga and ^67^Zn(p,n)^67^Ga nuclear reactions. Thus, a highly enriched ^68^Zn [25] and proton irradiation energy below 14 MeV can produce high ^68^Ga radioactivity with below limit ^66^Ga, and ^67^Ga [26].

Depending on the method, ^68^Ga production using cyclotrons can reach up to 37.5 GBq per production with a nominal proton energy of 12.5 MeV cyclotron [27]. The large ^68^Ga production would allow large-scale labeling of peptides. The problem of the possible occurrence of radiolysis can be solved by adding radical scavengers, such as sodium ascorbate and ethanol [28,29]. Furthermore, the apparent molar activity (AMA) which accounts for the amount of radioactivity per unit mole of non-radiolabeled, radiolabeled impurities, and remaining precursor for cyclotron production, is on par with or improved from the ^68^Ge/^68^Ga generator production [30,31]. The progress in ^68^Ga radionuclide production with cyclotrons encourages further studies that could lead to the establishment of a routine route for ^68^Ga-labeled peptide preparation. 

This review discusses the production of ^68^Ga with cyclotron using solid and liquid targets and the prospects and issues associated with cyclotron production. 

## 2. Cyclotron-Produced ^68^Ga Using a Solid Target

^68^Ga can be produced from solid targets using galvanized, pressed, foil, or molten targets. The irradiation parameters discussed were based on Advanced Cyclotron Systems, Inc. (ACSI) Richmond, Canada or GE Healthcare, Milwaukee, WI, USA cyclotrons. The introduction of pneumatic transfer systems, such as the QUANTM Irradiation System™ (Vancouver, Canada), Solid Target Transfer ACSI (Richmond, Canada), and ALCEO Solid Target Processing System, COMECER (Castel Bolognese, Italy) was a critical development for ^68^Ga production using solid target [32]. After the target irradiation, the target material is transferred to hot cells for dissolution with either concentrated hydrochloric acid (HCl) or nitric acid (HNO_3_). The separation of target material, purification, and formulation utilizing one-, two-, or three-column methods, making it possible to obtain high AMA in the shortest time at the end of purification (EOP) [22,32,33,34,35,36,37,38,39].

### 2.1. ^68^Ga Production Using a Solid Target in a Medical Scale PETtrace Cyclotron (GE Healthcare, Milwaukee, WI, USA)

Previous experience with the GE PETtrace cyclotron has shown promising results [22,32,34,38,39], subsequently opening the possibility of distributing ^68^Ga radiopharmaceuticals to multiple centers. The setup shows a saturation yield and EOP of above 1 GBq/μA and 3.7 GBq, respectively, as displayed in Table 1. 

The highest yield at the end of bombardment (EOB) was more than 370 GBq [32,39]. Both studies recorded the same target material, current and nominal proton energy. However, Thisgaard and co-workers [32] used 28% more mass of enriched ^68^Zn and a longer irradiation time than Svedjehed and group [39]. This was subsequently reflected in an increase in end-of-purification (EOP) activity of 18.8 GBq (approximately 10% more yield). For smaller-scale production, Siikanen and co-authors [34] reported that their capacity to produce an EOB activity of 31 ± 1 GBq ^68^Ga with a respectable saturation yield of 2.48 ± 0.06 GBq/μA using a foil target with a dimension of 15.5 mm or a target mass of 40 mg, respectively. This method is advantageous in centers that lack electroplating equipment and expertise.

Although influenced by other variables, the electroplated target produces a higher yield than the foil target. As recorded by Lin and his colleagues [22] with an electroplated target, the EOB was 60.9 ± 1.8 GBq, and the saturation yield was 2.72 ± 0.08 GBq/μA. Meanwhile, as mentioned in the study with a foil target, Siikanen and group [34] achieved an EOB of 31 ± 1 GBq with a saturation yield of 2.48 ± 0.06 GBq/μA, albeit with about 34% more ^68^Zn target. It was suggested that using a platinum disc reduced metallic impurities, especially during dissolution, thereby improving the EOP yield. Tieu and others [38] found that an EOP of 3.7 ± 0.18 GBq [^68^Ga]GaCl_3_ could be achieved with a low target mass (35.3 ± 2.2 mg) and a shorter time (8.5 min). Furthermore, Lin and colleagues [22] reported that the co-production of ^67^Ga impurities was less than 0.2% which can be reduced if the nominal proton energy is decreased to below 14 MeV. Specifically, for PETtrace cyclotron, this can be achieved by modifying the thickness of the energy degrader.

### 2.2. ^68^Ga Production Using a Solid Target with ACSI Cyclotron

The ACSI cyclotron, namely TR-19 and TR-24, has the ability to provide a variable energy spectrum without entirely relying on an energy degrader. Alnahwi and group [33] and Nelson and group [27] studied the production of ^68^Ga using a solid target and the TR-19/TR-24 cyclotrons ACSI utilizing a pressed target, as presented in Table 2. To prepare the target, Alnahwi and co-workers used a few steps in which the target was pressed with 250 mg of enriched ^68^Zn at 17,600 psi for 5 min before being inserted into a magnetic target carrier [33]. On the other hand, Nelson and group [27] used 100 mg of hydraulically pressed, enriched ^68^Zn at 35 kN to produce a 400 μm thick pellet, which was then sintered at 350 °C for 5 h before being placed on a silver support. The target was then pressed with 20 kN at 120 °C for 30 s to achieve a target density of 3.18 g/cm3 [27]. 

Alnahwi and co-workers [33] used a 400 μm aluminum integrated degrader to decrease the proton beam from 17.2 MeV to 13-14 MeV on the target material, while Nelson and group [27] used a 250 μm silver degrader to decrease the proton beam from 17 to 12.5 MeV. The saturation yield (8.7 GBq/μA) produced by Alnahwi and co-workers was higher than that of other studies presented in this review. Remarkably, the mass of the enriched ^68^Zn in their experiment was only 3.39 mg to produce 1 GBq of ^68^Ga. Thus, this method may be the most cost-effective, as it uses only pressed targets, and an irradiation time of 30 min is required to produce 68.8 GBq.

Pressed targets are usually less time-consuming to produce. However, Nelson and their group found traces of ^107^Cd and long-lived ^109^Cd related to the activation of silver backing. Metallic impurities other than Gallium isotopes can be removed during the separation of target material and purification. Nevertheless, the co-production of ^66^Ga and ^67^Ga was less than 0.1% of total radioactivity 4 h post-irradiation. Furthermore, they validated the silver disc pellet for 10 irradiations without significant degradation, limiting the time required to prepare the silver disc [27].

### 2.3. Solid Target Dissolution, Target Material (^68^Zn) Separation and Purification, and ^68^GaCl_3_ Formulation

Following irradiation, the solid target is transferred using a pneumatic transfer system for dissolution. The dissolution of the target is another important aspect related to the final activity obtained, and in the case of the short half-life of ^68^Ga, the time required for purification and its AMA are crucial. Thus, an automated procedure for target material (^68^Zn) separation and purification, and [^68^Ga]GaCl_3_ formulation using one-, two-, and three-column strategies has been reported. The typical steps of preconditioning and loading, washing, and eluting with an appropriate solution are performed on each column. This is to achieve low-volume and low-molarity of [^68^Ga]GaCl_3_, which in turn results in either high AMA, similar or improved from post-processing of the ^68^Ge/^68^Ga generator eluate [40,41,42,43,44,45].

The dissolution of the solid target and purification of [^68^Ga]GaCl_3_ using TR-19 and TR-24 cyclotrons is presented in Table 3. The typical dissolution solution would be using a concentrated HCl of 7 M or more to form [^68^Ga]GaCl_3_. This is imperative to ensure maximum dissolution and the right molarity for optimized retention. In contrast, Alnahwi and co-workers used a different approach using 7 M HNO_3_ [33]. This proceeds by adjusting the pH using ammonium formate (NH_4_HCO_2_) to retain up to 97% of the EOB activity in the hydroxamate resin. The group also noted a dark red (ferric hydroxamate) on the top of the hydroxamate resin. They, therefore, recommended using 200–300 mg of hydroxamate resin and 2 mL of 0.75 M HCl as the eluent to limit the iron in the ^68^Ga solution. The collected ^68^Ga was then diluted with 8 mL of 0.01 M HCl, transferred into the cation exchange resin CUBCX123, and washed with 30 mL of 0.01 M HCl. The final [^68^Ga]GaCl_3_ was collected in 12.5 μL NaCl 5 M/HCl 5.5 N, with an AMA of 28.3 ± 6.8 GBq/μmol [33]. 

**Figure 3:**
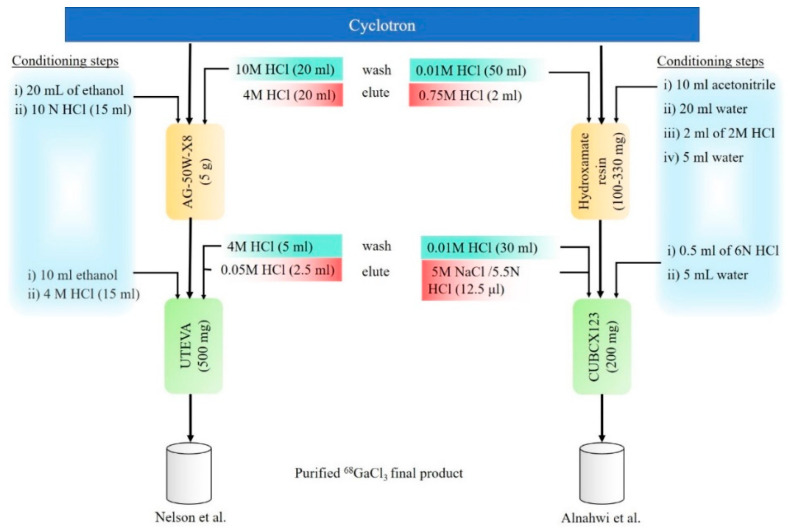
Summary of the steps in the purification of ^68^Ga from TR-24 and TR-19 cyclotron [27,33].

Nelson and colleagues [27] performed the dissolution method using 10 M HCl for 5 min. The column used for the separation of target material and formulation was different from Alnahwi and group [33], as depicted in Figure 3. The retention of ^68^Ga performed by Nelson et al. [27] was 5 g AG^®^ 50WX8 resin. The ^68^Ga was then transferred into a UTEVA column for formulation in 0.05 M HCl. Nonetheless, the method performed by Alnahwi et al. [33] resulted in higher AMA, mainly contributed by the low final volume of 12.5 μL of [^68^Ga]GaCl_3_.

The dissolution of a solid target from works using a GE PETtrace cyclotron is presented in Table 4. Thisgaard and co-workers heated concentrated HCl to 90 °C using the QIS Dissolution System to dissolve the target [32]. In contrast, Siikanen and group [34] dissolved the foil target in 9.5 M HCl for 2 min, the fastest of all methods in this review. Studies using single-column purification indicated respectable yields for peptide radiolabeling, which took less than 45 min [38,46]. The single-column strategy purifies ^68^Ga, separates ^68^Zn, and finally formulates to [^68^Ga]GaCl_3_ in 0.05 M HCl using octanol-based TK400 resin. Before elution, the column was washed with 0.7 mL of 0.05 M HCl to remove acid residues from 7 M HCl that could affect the [^68^Ga]GaCl_3_ eluate. The final volume and concentration of [^68^Ga]GaCl_3_ were 3.5 mL in 0.05 M HCl, corresponding to the generator eluate resulting in an AMA of 7.1 GBq/μmol. However, the group reported significant radiolysis in their study [38]. This may occur during radiolabeling or chelation reactions where radiosensitive precursors are degraded [47,48,49].

Other studies have used two-column methods with a strong cation-exchange column and UTEVA resin. Lin and co-workers utilized 4% cross-linking of 5 g AG 50W-X4 [22], while Nelson and his group used 8% cross-linking of 5 g AG 50W-X8 of a strong cation-exchange column [27]. The same solution was used in both studies, although Nelson and their group added ethanol when conditioning both columns. In contrast, Siikanen and his colleagues [34] eluted [^68^Ga]GaCl_3_ with 1 mL of water, producing the highest AMA of 86 ± 22 GBq/μmol. In this method, two UTEVA columns were used to purify and formulate [^68^Ga]GaCl_3_. [^68^Ga]GaCl_3_ was trapped with 4 M HCl, and both 4 M HCl and 2.5 M HCl were used for washing. The volume and residual acidity of the final [^68^Ga]GaCl_3_ may have contributed to the high AMA. The method contributed to an activity recovery of 76 ± 8% at EOP. The AMA in this work demonstrates the effect of highly concentrated [^68^Ga]GaCl_3_ at a presumably low acidic molarity [34]. Apart from Alnahwi and co-workers, the use of hydroxamate resin was also investigated by Thisgaard and group [32]. They used a three-column strategy with the addition of LN resin between hydroxamate (ZR resin) and TK200 resin. The specific role of LN resin in this setup was to trap excess iron impurities before ^68^Ga was retained in TK200 resin. The setup produced [^68^Ga]GaCl_3_ in 2.5 mL of 0.1 M HCl with an AMA of 25 GBq/μmol [32].

## 3. Cyclotron-Produced ^68^Ga Using a Liquid Target

Further developments in the production of ^68^Ga using medical cyclotrons have been attempted, with the most recent success with liquid targets [35,36,50,51]. These results will encourage the routine production of ^68^Ga, particularly in facilities unable to produce via solid targets. Currently, three notable works [35,36,37] use the PETtrace cyclotron described in Table 5. In contrast to the solid target, the ^68^Zn target was prepared in nitric acid ([^68^Zn]Zn(NO_3_)_2_) in the liquid form. Previous experience with irradiation of zinc salt, especially ZnCl_2_, reported gas formation that subsequently increased the pressure inside the target [52]. This reaction is caused by the radiolysis of water after ionizing radiation and forming radicals [53]. The presence of chloride ions further enhances gas formation, whereas nitrates induce the opposite. Thus, nitric salts are used to reduce gas formation, whereas the addition of nitric acid prevents the formation of a solid precipitate [35]. 

Table 6, Table 7 and Table 8 represents an overview of studies on cyclotron-produced ^68^Ga with a liquid target. Riga and co-workers [35] produced 4.3 GBq ± 0.3 ^68^Ga activity using 1.7 M [^68^Zn]Zn(NO_3_)_2_ in 0.2 M nitric acid after 32 min of irradiation at 46 µA. More than 75% of the initial activity was retained during post-processing. Pandey and group [36] reported a higher EOB of 9.85 ± 1.6 GBq using 1.42 M [^68^Zn]Zn(NO_3_)_2_ in 1.2 M nitric acid as starting material when irradiated with 40 µA for 60 min as described in Table 6. The group further experimented with the effect of [^68^Zn]Zn(NO_3_)_2_ concentration and irradiation time on yield activity [51,53]. They found that reducing the [^68^Zn]Zn(NO_3_)_2_ concentration to 0.6 M would decrease the yield to 3.94 GBq ± 0.20. Moreover, shortening the irradiation time to 30 min and lowering the beam current to 30 µA reduces the activity, as 0.6 M [^68^Zn]Zn(NO_3_)_2_ only produces 1.64 GBq ± 0.07 of ^68^Ga, whereas 1.2 M [^68^Zn]Zn(NO_3_)_2_ produces 3.37 GBq ± 0.17 of ^68^Ga at EOB. In addition, the lower yield owing to the longer irradiation time could be avoided by increasing the nitric acid concentration. This effect was mainly due to the high nitric acid consumption at a long irradiation time (<30 min) and high beam current. Nonetheless, increasing the nitric acid concentration can potentially damage the materials connected to the target. Thus, it is important to properly evaluate the equipment and materials used to potentiate high concentrations of nitric acid in the preparation of the target material [51,53].

The irradiated [^68^Zn]Zn(NO_3_)_2_ was then transferred to a synthesizer for separation of target material and purification to form [^68^Ga]GaCl_3_ as described in Table 7. The separation of target material and purification processes were performed using a two-column method: (1) ZR resin and (2) TK200 resin or AG-1X-8 anion exchange resin. The ZR resin was conditioned with 0.1 M HNO_3_ before trapping. After ^68^Ga trapping, the ZR resin was washed with 0.1 M HNO_3_ and then eluted with 1.75 M–2 M HCl to TK200. This method produces 5 mL [^68^Ga]GaCl_3_ in 0.1 M HCl. Rodnick and co-workers [37] highlighted a clear difference in using TK200 resin. The group introduced the NaCl/HCl purge method to reduce the residual acid in the final [^68^Ga]GaCl_3_ formulation of 0.1 M HCl in 5 mL [37]. Pandey and co-workers had a different approach whereby the pH of target solution was adjusted to 5.5–6.0 using sodium bicarbonate to improve the retention of ^68^Ga. In addition, an AG-1X-8 anion exchange resin was used for formulation and only 2 mL water was needed to elute the [^68^Ga]GaCl_3_ for radiolabeling [36]. Both methods were important to ensure a high AMA, especially when using a liquid target, thus reducing the impact of a lower EOB compared to a solid target.

The radiolabeling proceeds with elevated temperature and a typical pH of 4.0 to 4.5 as presented in Table 8. Pandey and co-workers were able to obtain [^68^Ga]Ga-PSMA-11 EOS of 1.78–3.16 GBq, whereas Rodnick and group produced 1.67 GBq [36,37]. The differences in EOS published by both authors were largely contributed to the EOB activity as presented in Table 6.

Some areas can be added to improve the EOS of ^68^Ga produced using liquid targets. Al-Nahwi and colleagues [33] found that the pH of ^68^Ga in nitric acid influenced trapping in hydroxamate resin. More than 97% of the activity was retained at a pH above 2. However, at pH 3 and above, the activity that persisted in the dissolution vial was high. Although this study was performed using a solid target, it would be valuable if it was implemented for a liquid target. Moreover, the extension of the irradiation time to more than 60 min should be studied further. Pandey and co-workers noted the influence of nitric acid concentration in ensuring a high saturation yield [36]. However, this should depend on the material used because a high nitric acid concentration could damage targets and possibly cyclotrons, which would necessitate frequent target maintenance [32]. 

## 4. Matters in ^68^Ga Cyclotron Production

The promising future for cyclotron production of ^68^Ga will increase the availability of such radiopharmaceuticals, especially parallel to the number of medical cyclotrons reported by the International Atomic Energy Agency (IAEA). However, a few considerations should be considered to ensure consistent production and sustainable supply of cyclotron-produced ^68^Ga radiopharmaceuticals [54,55,56].

### 4.1. Expansion of Solid Target ^68^Ga Preparations

The production of radionuclides with solid targets requires technical skills and knowledge, such as target preparation, irradiation, target transport, and dissolution methods. Recent developments, such as the automated target transport and dissolution system allow minimal work, consequently reducing radiation exposure and improving product consistency.

For future expansion, a solid [^68^Zn]ZnO target should be commercially available to complement the advances in automated target transport and dissolution systems. Experience suggests that the electroplated target offers a higher EOB yield with less [^68^Zn]ZnO than the foil and pressed target, despite the long and tedious procedure. This would be advantageous for central nuclear pharmacies only if the cost of the commercially available electroplated [^68^Zn]ZnO target was economical. In addition, cassette-based synthesis should be readily available for cost efficiency and reliable production, which minimizes the influence of human error and enables more consistent production [57].

### 4.2. Sustainable Practice in Cyclotron-Produced ^68^Ga: [^68^Zn]ZnO Target Reprocessing

The cost of upgrading the cyclotron to produce ^68^Ga radiopharmaceuticals for liquid targets appears to be much lower than that for solid targets, allowing access to and continuity of service, especially in developing countries [58]. Priority should be given to reprocessing the recovered ^68^Zn target to maximize resource use and increase cost-effectiveness. The investigation of the reprocessing of ^68^Zn targets is presented in Table 9. Pandey and group [36] and Riga and group [35] reprocessed the recovered ^68^Zn with purities up to 99%, the former using a cation exchange resin (AG-50WX8) and the latter using a drying method. Only minor impurities were detected which shows that reprocessing the recovered ^68^Zn target is possible with minimal labor. Further experiments should be performed to evaluate the quality of the irradiated reprocessed ^68^Zn.

Metal impurities in the [^68^Zn]ZnO target affect the EOB and the final product, especially if ^66^Ga and ^67^Ga are present. Several precautions should be taken to prevent metal contamination of reprocessed [^68^Zn]ZnO during pre-processing, processing, and post-processing. In general, the use of metals should be avoided, including contact with metal equipment. As described in Table 10, pre-processing precautions include using trace metal-free water and HNO_3_ specifically in the production of [^68^Zn]ZnO and any chemicals that come into contact with [^68^Zn]ZnO during production. To recover [^68^Zn]ZnO, a clean, sterile vial with a coated or plastic needle is preferred.

The presence of ^67^Zn may have arisen as a by-product of the ^68^Zn(p,2n)^67^Ga reaction. Although this is difficult to determine because ^67^Zn may be present only in minimal amounts, any change in the production process or any low yield result should be considered for the possibility of ^67^Zn presence. 

During the processing of the recovered [^68^Zn]ZnO, it is important to take preventive measures, such as using trace metal-grade water and chemicals for dilution and equipment cleaning. The equipment used should also be dedicated to preventing cross-contamination with materials that could affect the reprocessed [^68^Zn]ZnO quality. The reprocessed [^68^Zn]ZnO was stored in an airtight container. For validation purposes, both inductively coupled plasma mass spectrometry (ICP-MS) and gamma spectrometry analyses were performed for three consecutive runs. In the case of multiple [^68^Zn]ZnO reprocessing, a risk assessment and appropriate study should be considered. This may have regulatory implications, such as the potential cause of cross-contamination with other metals.

### 4.3. Optimization of ^68^Ga Radiopharmaceutical Production via a Liquid Target

The production of ^68^Ga radiopharmaceuticals via a cyclotron is touted as “production on demand,” given that it can be produced with a consistent EOS activity at any time upon need throughout the year. This development may create more opportunities for cyclotron centers to upgrade for ^68^Ga production. Installing a solid target may be costly; hence, the liquid target is the better option, especially for medical cyclotrons. Nonetheless, the liquid target production of ^68^Ga radiopharmaceuticals has more room for optimization to ensure cost efficiency for each production. This can be deduced as improvements in EOS.

The ideal characteristics of a cyclotron-produced ^68^Ga radiopharmaceutical include (1) a short production time, (2) minimal ^68^Ga losses during purification, and (3) a high AMA. Due to the short half-life of ^68^Ga, the purification and radiolabeling steps must be rapid to achieve a high yield at EOS. However, using two-column methods for purification and formulation became an obstacle to shorter preparation time. Further work can be considered for purification and formulation using liquid target production to reduce time and improve EOS.

## 5. Regulatory Aspects of Cyclotron-Produced ^68^Ga Radiopharmaceuticals

Recently, the direct production of cyclotron-based ^68^Ga, particularly the proton irradiation of enriched ^68^Zn target route, has expanded and been practiced elsewhere. Currently, most medical cyclotron produces ^68^Ga radiopharmaceuticals to cater for in-house use, which does not require marketing authorization (MA) from regulatory bodies. To produce medicinal products for human use, each country follows a set of national or international guidelines that are being enforced by local regulatory bodies, such as the US Food and Drug Administration (US FDA), World Health Organization (WHO), the International Conference on Harmonization of Technical Requirements for Registration of Pharmaceuticals for Human Use (ICH), or Pharmaceutical Inspection Co-Operation Scheme (PIC/S) [59]. 

Producing radiopharmaceuticals compound may be categorized under Good Manufacturing Practices (GMP) or Good Preparation Practices (GPP). The main differences between GMP and GPP are summarized in Table 11. The nature of cyclotron-produced ^68^Ga radiopharmaceuticals falls under the grey area. Although the criteria mostly fit the elements listed under GMP, the radioisotope’s short half-life limits the labeled compound’s distribution to other centers or institutions.

As mentioned, most medical cyclotron facilities produce ^68^Ga radiopharmaceuticals for in-house use only; thus, it is considered a small-scale preparation. EANM recently released a Guideline on the current Good Radiopharmacy Practice (cGRPP) for the small-scale preparation of radiopharmaceuticals to cater to in-house radiopharmaceutical preparations [60]. The cGRPP guidance was not much different from the PIC/S GPP guidance document practices elsewhere. EANM position for in-house radiopharmaceutical preparation also clearly stated that MA is not mandatory [61]. 

However, using a solid target to produce ^68^Ga radiopharmaceuticals may yield a different opinion from manufacturers and regulators. The capacity to produce ^68^Ga radiopharmaceutical through a solid target using a medical cyclotron can be considered a large-scale production with a range of minimum EOP and EOB between 3.7 to 18 GBq and 6.3 to 31 GBq, respectively [34,38]. With this range of [^68^Ga]GaCl_3_ produced, the ^68^Ga radiopharmaceutical could be distributed to other centers. This scenario may require MA approval from local authorities, which requires a GMP license to produce the dedicated radiopharmaceuticals as well as registration of products. 

There are a few options on how cyclotron-based ^68^Ga radiopharmaceuticals can be supplied either as [^68^Ga]GaCl_3_ or as a labeled radiopharmaceutical such as [^68^Ga]Ga-DOTA-TATE or [^68^Ga]Ga-PSMA. The nature of the ^68^Ga radiopharmaceutical also significantly determines the need for MA approval. Generally, medicinal products required to be registered and obtain MA are intended for human use to diagnose, cure, treat or prevent any ailment or disease. Thus, [^68^Ga]GaCl_3_ does not fit the definition of the medicinal product, unlike [^99m^Tc]TcO^4−^ in pertechnetate, from where it can be given directly for thyroid scintigraphy. The [^68^Ga]GaCl_3_ cannot be administered in free form unless it is radiolabeled with a precursor, such as DOTA-TATE and PSMA. In this case, [^68^Ga]GaCl_3_ needs to be supplied as an active pharmaceutical ingredient requiring full quality testing and documentation for approval by regulatory bodies. 

Besides that, there are also arguments that the labeled [^68^Ga]Ga-DOTA-TATE or [^68^Ga]Ga-PSMA does not fit the manufacturing elements as the nature of the production process is more towards reconstitution instead of manufacturing. The radiolabeling process is the same as the one generator produced except that cyclotron-produced ^68^Ga needs additional steps, such as processing of the solid target and purification steps before it is in the form of ready-to-label [^68^Ga]GaCl_3_. These additional steps are critical and will determine the fate of the final product; therefore, it is considered a high-risk preparation that fits the manufacturing element. 

When considering GMP for cyclotron-based ^68^Ga radiopharmaceuticals, a few parameters need to be considered starting from target transfer and processing, chemical preparations, synthesis module software, process validation, quality control testing, and metal testing. These parameters will be discussed briefly in the following subsection.

### 5.1. Target Transfer System and Processing

Under PIC/S GMP Guide Annex 3 (manufacture of radiopharmaceuticals) stated that the GMP requirement is not mandatory for the cyclotron. However, the cyclotron and its transfer system may consider the first steps of active substance manufacture, which require the process to be included under the GMP element [62]. The performance qualification (PQ) for the cyclotron liquid target is straightforward and easily implemented. Cyclotron target PQ shall include ^68^Ga EOB activity, radionuclidic purity, percentage of activity lost, and volume test. For solid target production, additional steps are required where the solid target needs to be dissolved in an acidic solution, such as HCl or HNO_3_; only then can it be further purified using single or multiple resins. There are a few options on how the dissolution process can be performed: (1) process within the cyclotron vault (in-situ); (2) process in a dedicated hot cell; and (3) process within the same hot cell where the synthesis module is located (integrated with synthesis module [39,63,64]. Option (1) and (3) are the most suitable for medical-grade cyclotron as such a system does not require a dedicated hot cell for the dissolution process while providing automation that reduces unnecessary radiation exposure for operators. From a GMP point of view, these processes need to be validated and included in the PQ for a solid target system. Additional testing parameters for the equipment qualification shall be considered for heating verification, consistency in the volume of acid for the dissolution process, and time verification for the whole solid target transfer and dissolution process.

### 5.2. Chemical Preparations

Generally, cyclotron-based ^68^Ga production employs manual chemical preparations versus commercial cassettes, as the latter is costly and is not widely available. Preparing cyclotron-based ^68^Ga radiopharmaceuticals using a commercial cassette may have advantages in terms of simplicity and GMP compliance. There is no need to perform validation for chemical components except for raw material sampling, which can be conducted initially for vendor qualification purposes. The procedure needs to be validated for manual chemical preparation, and the preparation must be completed aseptically under controlled environments. The laboratory apparatus and glassware must be calibrated. Furthermore, the glassware cleaning procedures shall also be validated to avoid cross-contamination, which will affect the integrity of chemical preparation. The most critical point to be considered is the random sampling of the manually prepared chemicals for sterility and endotoxin limits that need to be established for each lot.

### 5.3. Synthesis Module Supervision Software

The synthesis module relies on the software to control the equipment’s specific operation and capture production data. Under PIC/S GMP Annex 11, all applications and IT infrastructure related to production equipment or process must be qualified. However, the software for the synthesizer platform is categorized as configurable software where the Programmable Logic Controller (PLC) is built-in on the equipment. The software is limited to controlling specific operations and functions; thus, complete validation and qualification are not mandatory to be performed by the user. The manufacturer shall comply with Good Automated Manufacturing Practice 5 (GAMP 5) during the development of the synthesis module software. The software validation certificate shall be issued during Installation Qualification (IQ). The only concern is the synthesis sequence that can be manipulated depending on needs and situations. Unlike [^18^F]Fluorodeoxyglucose (FDG) manufacturing, the synthesis sequence and software are fully qualified by the manufacturer, where a Common Technical Document (CTD) is provided that describes the technical aspects of manufacturing and regulatory support information. Meanwhile, for ^68^Ga manufacturing, the synthesis sequence is categorized as ‘open-sequence’ where the parameter can be adjusted/interrupted during the synthesis, which may or may not be a concern for regulators. Nonetheless, some software platforms can limit operator control by password, plus the software will capture any manual interruptions during synthesis, and the information is stated in the production report.

### 5.4. Quality Control of ^68^Ga Radiopharmaceuticals

Since the monograph for Accelerator Produced [^68^Ga]GaCl_3_, [^68^Ga]Ga-DOTA-TATE, and [^68^Ga]Ga-PSMA are available, there should be no issue in performing the QC test accordingly. Strict validation should be performed, in particular for co-produced ^66^Ga and ^67^Ga impurities, since the testing is performed up to 24 h after the production. Though the production of radiopharmaceuticals is not alienated from performing tests after administration to patients, typically for sterility tests, it is vital to ensure that the output is within the specifications. Hence, these validation data should be compiled, and the routine production should not deviate from the standard procedure. These risks should also be considered in low EOB or EOS cases. In addition, an impurity detection test should be validated and performed considering the risk of cross-contamination, mainly if different ^68^Ga radiopharmaceuticals are produced within the same day.

### 5.5. Metal Testing for [^68^Ga]GaCl_3_

The metal testing result is required for [^68^Ga]GaCl_3_ but not mandatory for a labeled compound. The metal that needs to be assessed is Zn and Fe, where the limits are 10 µg/GBq after EOP as stated in the EU monograph. Most medical cyclotron facilities do not equip with a metal testing device as such equipment is costly, and the test is limited to one test only, thus will result in low usage. Since the metal test for [^68^Ga]GaCl_3_ does not require the sample to be tested immediately, the sample can decay out in a proper container or vial and be sent to a third-party laboratory for metal testing. The test is required each time [^68^Ga]GaCl_3_ has been produced. Thus, stages of product release shall be employed and stated clearly in the procedure. Furthermore, a contractual agreement shall be in place with a third-party analytical lab that details the manufacturer’s and analytical lab’s scope and role. These elements shall be considered if the manufacturer intends to obtain a GMP license.

### 5.6. Process Validation

Process validation, including media fill validation that exposes microbial growth media to the product contact surface, equipment, or container closure system mimics the actual production, shall be performed periodically as a requirement of sterile product manufacturing. In the synthesizer platform, the media cannot replace the chemicals and reagents as it is impossible to perform synthesis runs using microbial growth media such as Tryptic Soy Broth (TSB) that cannot pass through the small tubing and cassette due to the media thickness. A proper media simulation validation protocol shall be designed accordingly not to compromise the main objectives of process validation. One can consider diluting the media with water for injection (WFI). Still, the dilution ratio needs to be validated to ensure that it can promote microbial growth with the diluted media.

### 5.7. Other Consideration

Since the cyclotron can produce ^68^Ga radiopharmaceutical on a large scale, other options can be considered to utilize the labeled compounds fully. Typically, most nuclear medicine facilities are equipped with one PET; thus, producing a large-scale ^68^Ga radiopharmaceutical is not worthwhile because the ^68^Ga scans are limited to the number of FDG scans. To maximize the utilization of large-scale ^68^Ga production, other patients from nearby non-cyclotron PET centers within the same district or area can perform injections on-site and the scans at their facility. The idea is to provide a new clinical service instead of supplying radiopharmaceuticals to other centers needing MA approval. The advantage of this innovative service is that the patient preparation for ^68^Ga scans is not as tight as FDG scans; thus, the patient can be ready for injection once the synthesis is completed, and the QC results are within the specification. Nevertheless, some limitations must be considered, such as transporting ‘radioactive’ patients that may require approval from local authorities.

## 6. Conclusions

In addition to the significant clinical benefits, radioisotope supply sustainability is crucial for meeting current and future needs. The cost and availability of radioisotopes are critical factors in ensuring a future-proof supply. Though alternatives to ^68^Ga, such as [^18^F]AlF radiopharmaceuticals, are being intensively discussed [65,66,67,68], the narrative for theranostic ^68^Ga/^177^Lu for PRRT remains critical through the work on the expansion of the production route, as reviewed in this article. With the increasing use of ^68^Ga in nuclear medicine and the introduction of new ^68^Ga radiopharmaceuticals, ^68^Ga availability is becoming increasingly critical. Therefore, the innovative cyclotron method for producing ^68^Ga is touted as a significant advancement in this field, particularly because it allows for a very high ^68^Ga yield. The innovative approach described in this article to producing liquid targets will soon be feasible, subject to the approval of local authorities. The producers of enriched [^68^Zn]ZnO from the local region and validated/automated for reprocessing [^68^Zn]ZnO could later ensure the cost-effectiveness of ^68^Ga produced from the cyclotron.

## Figures and Tables

**Table 1:** ^68^Ga production with PETtrace Cyclotron with ^68^Zn irradiation. Included yield activity at EOB and EOP.

Target Preparation	Nominal Proton Energy	Irradiation Parameters	EOB (GBq)	Specific Activity (GBq/μg)	Saturation Yield (GBq/μA)	EOP (GBq)	Ref.
Target material: Electroplated on a platinum disc Dimension: 7.0 mm ^68^Zn mass: 104.1 ± 2.7 mg	14.5 MeV	Current: 30 μA Time: 60 min	60.9 ± 1.8	NR	2.72 ± 0.08	NR	[22]
Target material: Electroplated on a platinum backing Dimension: 10.0 mm ^68^Zn mass: 35.3 ± 2.2 mg	14.5 MeV with 320 μm aluminium degrader foils	Current: 35 μA Time: 8.5 min	6.3 ± 0.4	2530	1.26 ± 0.08	3.7 ± 0.18	[38]
Target material: Electroplated water-cooled silver backing Dimension: 10.0 mm ^68^Zn mass: 300 mg	13.0 MeV on target. helium-cooled aluminium foil energy degrader	Current: 80 µA Time: 120 min	>370	NR	NR	194	[31]
Target material: Foil Dimension: 15.5 mm ^68^Zn mass: ~140 mg	12.6 MeV with 500 μm aluminium energy degrader	Current: 25 μA Time: 68 min	31 ± 1	1209 ± 18	2.48 ± 0.06	18 ± 2	[34]
Target material: Electroplated on a silver backing Dimension: ~10.0 mm ^68^Zn mass: 216 ± 10 mg	13.0 MeV with 500 μm aluminum foil degrader	Current: 80 μA Time: 102 min	370	NR	7.1	175.2	[39]

NR—Not reported.

**Table 2:** ^68^Ga production using solid target on an Advanced Cyclotron Systems, Inc. (ACSI) Cyclotrons.

Cyclotron	Pressed Target Preparation	^68^Zn Mass (mg)	Nominal Proton Energy (MeV)	Irradiation Parameter	Saturation Yield (GBq/μA)	EOB (GBq)	% Total ^67^Ga and ^66^Ga post 6 h	Ref.
TR-19	^68^Zn powder (ISOFLEX, San Francisco, CA, USA) Dimension: ~10.0 mm Thickness: 0.55 mm Density: 1.43 g/cm^3^	247	14	Current: 30 μA Time: 30 min Proton beam energy: 17.2 MeV	8.7	68.8 ^a^	<2	[33]
TR-24	^68^Zn powder (ISOFLEX, CA, USA) Dimension: ~10.0 mm Thickness: 0.40 mm Density: 3.18 g/cm^3^	100	12.5	Current: 30 μA Time: 73 min ^b^ Proton beam energy: 17 MeV	2.4 ± 0.12	37.5 ± 1.9	0.51	[27]

^a^ Taken from entry number 5; ^b^ Taken from the second experiment where irradiation was 2200 μA·min.

**Table 3:** Dissolution of solid target for TR cyclotron and purification of [^68^Ga]GaCl_3_.

Target Dissolution	Separation of Target Material and Purification	Formulation	Time (min)	EOP (GBq)	[^68^Ga]GaCl_3_ Molarity (Volume)	EOS (GBq)	AMA (GBq/µmol)	Ref.
S: 7 M HNO_3_ V: 1–2 mL Additional: adjust pH using NH_4_HCO_2_ (2–2.5 mL, 2.5 M) to pH 2	R: Hydroxamate (200–330 mg) C: acetonitrile (10 mL), water (20 mL), 2 M HCl (2 mL), water (20 mL) W: 0.01N HCl (50 mL) E: 0.75 N HCl (2 mL)	R: CUBCX123 C: 0.5 mL 6 N HCl, 5 mL waterL: pre dilution using 0.01 M HCL (8 mL) W: 0.01 N HCl (30 mL) E: NaCl 5 M/HCl 5.5 N (12.5 μL)	<12	NR	5 M NaCl/5.5 M HCl (12.5 μL)	4.6 ± 0.1 ^a^	28.3 ± 6.8	[33]
10 M HCl in 5 min	R: 5 g of AG^®^ 50WX8 resin C: 20 mL of ethanol, 15 mL of 10 M HCl W: 20 mL of 10 M HCl E: 20 mL of 4 M HCl	R: 500 mg of UTEVA resin C:10 mL of ethanol, followed by 15 mL of 4 N HCl. W: 5 mL of 4 N HCl E: 2.5 mL of 0.05 M HCl	<30	37.5 ± 1.9	0.05 N HCl (2.5 mL)	NR	9.5 ± 1.3	[27]

S: Solution; V: Volume; T: Time; R: Resin; C: Conditioning; L: Load W: Wash; E: Elution; NR: Not reported. ^a^ 20 min irradiation at ~5 μA and Ep = 14 MeV.

**Table 4:** Dissolution of solid target for GE PETtrace cyclotron and purification of [^68^Ga]GaCl_3_.

Target Dissolution	Separation of Target Material and Purification	Formulation	Time (min)	EOP (GBq)	[^68^Ga]GaCl_3_ Molarity (Volume)	EOS (GBq)	AMA (GBq/µmol)	Ref.
S: 7 M HCl V: 0.5 mL	R: TK400 resin C: 7M HCl (5 mL) W: 7 M HCl (28 mL), 0.05 M HCl (0.7 mL) E: 0.05 M HCl (3.5 mL)	NA	32 ^a^	3.31	0.05 M HCl (3.5 mL)	1.56 ^b^	7.1	[38]
S: 10 M HCl V: 10 mL T: <10 min	R: 5 g of 50W-X4 C: 10 M HCl (25 mL) W: 10 M HCl (30 mL) E: 4 M HCl (12 mL)	R: 100 mg UTEVA^®^ C: 4 M HCl (10 mL) W: 4 M HCl (10 mL) E: 0.05 M HCl (2 mL)	10	NR	0.05 M HCl (2.0 mL)	NR	6.7 ± 0.8	[22]
S: 9.5 M HCl V: 2 mL T: 2 min	R: UTEVA L: Pre-dilution4 M HCl (11.5 mL) W: 10 mL of 4 M, HCl and 10 mL of 2.5 M HCl E: 2 mL water and re-distributed into 10 mL of 4 M HCl	R: UTEVA W: 20 mL of 2.5 M HCl E: 1 mL water	23	18 ± 2	Water (1 mL)		86 ± 22 ^c^	[34]
S: 30% HCl (~90 °C) V: 2 mL	R: 250 mg ZR resin. W:15 mL of 30% HCl E: 8 mL of 1 M HCl and passed through a LN Resin	R: TK200 resin. W: Nitrogen purging E: 2.5 mL of 0.1 M HCl	35	194 ^d^	0.1 M HCl (2.5 mL)	72.2	25 ^e^	[32]

S: Solution; V: Volume; T: Time; R: Resin; C: Conditioning; L: Load W: Wash; E: Elution; NR: Not reported. ^a^ From dissolution until end of purification; ^b 68^Zn target (23 mg) was irradiated at 14.5 MeV and 30 mA for 15 min to give 6.30 GBq; ^c^ DOTA; ^d^ Production run number 4; ^e^ DOTA.

**Table 5:** Target preparation or modification.

Internal Volume	Degrader	Support	Ref.
2.5 mL	250 µm foil of niobium liquid niobium-body target	25 µm Havar^®^ foils for helium cooling chamber and the helium cooling	[35]
2.0 mL	Dual foils of 200 µm aluminum	Havar (40 µm) separated by helium cooling.	[36]
2.2 mL	200 µm aluminum foil	25 μm Havar foil for support and 25 μm niobium foil for chemical inertness with the target media	[37]

**Table 6:** Published articles related to the production of cyclotron ^68^Ga.

Process	Method	Result	Ref.
Target material preparation and irradiation using GE PETtrace 800 cyclotron	Target: 1.7 M [^68^Zn]Zn(NO_3_)_2_ in 0.2 M HNO_3_ Current: 46 µA Time: 32 min Nominal proton energy: 12 MeV	EOB = 4.3 ± 0.3 GBq	[35]
	Target: 1.42 M [^68^Zn]Zn(NO_3_)_2_ in 1.2 M HNO_3_ Current: 40 µA Time: 60 min Nominal proton energy: 14 MeV	EOB = 9.85 ± 1.6 GBq (266 mCi)	[36]
	Target: 1.0 M [^68^Zn]Zn(NO_3_)_2_ in 0.2–0.3 M HNO_3_ Volume: 2.2 mL Current: 30 μA Time: 60 min Nominal proton energy: 14.3 MeV	EOB = 3.7 GBq (100 mCi)	[37]

**Table 7:** Methods for separation of target material and purification and formulation of [^68^Ga]GaCl_3_.

Process	Method	Result	Ref.
Separation of target material and purification, and formulation	Platform: FastLab2 Developer, GE Healthcare, Wisconsin, USA Purification: Zr Resin washed with 0.1 M HNO_3_ (9 mL), elute with 2 M HCl (5 mL) Formulation: TK200, elute with 0.1 N HCl (5 mL)	EOS = 2.3 ± 0.2 GBq	[35]
	Platform: Trasis All-in-One, Belgium Purification: 100 mg, hydroxamate resin (50–100 μm); washed with of 0.005 M HNO_3_ (50 mL); elute with of 5.5 M HCl (7 mL). Formulation: 400 mg, AG-1X-8 anion exchange resin; elution with 2 mL of water.	NR	[36]
	Platform: FastLab2 Developer, GE Healthcare, Milwaukee, WI, USA Purification: Zr Resin; condition with 0.1 M HNO_3_ (7 mL); washed with 0.1 M HNO_3_ (15 mL), elution with 1.75 M HCl (5–6 mL). Formulation: TK200 resin; condition with water (7 mL) followed by 1.75 M HCl (4 mL) before use; washed with 2.0 M NaCl (3.5 mL) in 0.13 M HCl; elute with 1–2 mL H_2_O followed by dilute HCl to formulate	EOS= 2.0 ± 0.3 GBq 50 mCi	[37]

**Table 8:** Method for [^68^Ga]Ga-PSMA-11 radiolabelling and the EOS radioactivity.

Process	Method	Result	Ref.
Radiolabeling	100 °C for 10 min at pH 4.0–4.5	[^68^Ga]Ga-PSMA-11 1.78–3.16 GBq (48.1–85.5 mCi, uncorrected)	[36]
	50 °C for 5 min	[^68^Ga]Ga-PSMA-11 were near quantitative (~1.67 GBq, 45 mCi)	[37]

**Table 9:** Method, yield, and ^68^Zn quality of ^68^Zn target reprocessing.

Method	Yield and ^68^Zn Quality	Ref.
Adjust the pH of the recovered ^68^Zn to ≤5 and use dilute nitric acid. Condition the cation exchange resin (AG-50WX8) with 60 mL water and 20 mL air. Load the recovered ^68^Zn onto the AG-50WX8 resin and wash with 20 mL of air, followed by 10 mL of water and 20 mL of air. Elute ^68^Zn with 15 mL of 8 M HNO_3_. Remove HNO_3_ under vacuum at 40 °C for 30 min and then at 60 °C. Freeze-dry overnight to remove residual water.	Yield: 82.6% ± 13.6 Purity: 99.5% Impurities: 0.5% Na as NaNO_3_	[36]
Heat the recovered ^68^Zn to dryness. Dissolve the remaining ^68^Zn with 6 M HNO_3_ and heat again until dry. Redissolve with 0.2 N HNO_3_.	Not available	[35]

**Table 10:** Precaution measures to be considered for [^68^Zn]ZnO reprocessing.

Step	Precaution Measure
Pre-processing	Use ultra-trace metal grade water to prepare the [^68^Zn]ZnO target. Use trace metal grade HNO_3_ to prepare for [^68^Zn]ZnO target. Avoid any contact with metal during the preparation of the [^68^Zn]ZnO target. Ensure that the chemicals used to capture and purify ^68^Ga are trace metal grade. Collect the ^68^Zn recovery in a clean, sterile vial. Never use a metal needle to collect the ^68^Zn recovery. Clean the target with trace metal grade HNO_3_. Ensure that the proton irradiation energy does not co-produce ^67^Ga via the ^68^Zn(p,2n)^67^Ga route.
Processing	Use ultra-trace metal water. Use trace metal analysis grade of HNO_3_. Use clean equipment cleaned with ultrapure water. Minimize any contact with metallic equipment for drying or removing moisture.
Post-processing	Keep the reprocessed [^68^Zn]ZnO in an airtight container, preferably in a vial. Perform isotopic analysis, preferably using ICP-MS, to detect ^66^Zn and ^67^Zn. Perform cyclotron irradiation of the reprocessed [^68^Zn]ZnO and analyze it with a gamma spectrometer to detect any co-production of ^68^Ga. Perform three consecutive validation runs using the same production sequence

**Table 11:** Main differences between Good Manufacturing Practices (GMP) and Good Preparation Practices (GPP).

Good Manufacturing Practice (GMP)	Good Preparation Practice (GPP)
Guidance for Industry on Manufacture of Medicinal Products	Guidance for Healthcare Establishments on the Preparation of Medicinal Products
Distribution of manufactured radiopharmaceuticals to local/international market	Direct supply to patient for in-house use
Manufacture in large-scale of radiopharmaceuticals	Preparation in a small scale of radiopharmaceuticals according to prescription
Starting from raw materials to finished products	Products are being prepared in dilution or reconstitution and/or mixing of products
High-Risk Preparation	Low-Risk Preparation
Distribution of radiopharmaceuticals needs to obtain Marketing Authorization (MA)	Distribution of radiopharmaceuticals does not need for Marketing Authorization (MA)
Full quality control test as per monograph	Partial quality control test based on manufacturer recommendation or in-house method

## Data Availability

Not applicable.
